# Loci Encoding Compounds Potentially Active against Drug-Resistant Pathogens amidst a Decreasing Pool of Novel Antibiotics

**DOI:** 10.1128/AEM.01438-19

**Published:** 2019-11-14

**Authors:** Joseph Basalla, Payel Chatterjee, Elizabeth Burgess, Mahnur Khan, Emily Verbrugge, Daniel D. Wiegmann, John J. LiPuma, Hans Wildschutte

**Affiliations:** a Department of Biological Sciences, Bowling Green State University, Bowling Green, Ohio, USA; b Department of Pediatrics, University of Michigan Medical School, Ann Arbor, Michigan, USA; Kyoto University

**Keywords:** *Pseudomonas*, antagonistic, antibiotic, biosynthetic gene cluster, multidrug resistance, transposon mutagenesis

## Abstract

Since the discovery of penicillin, microbes have been a source of antibiotics that inhibit the growth of pathogens. However, with the evolution of multidrug-resistant (MDR) strains, it remains unclear if there is an abundant or limited supply of natural products to be discovered that are effective against MDR isolates. To identify strains that are antagonistic to pathogens, we examined a set of 471 globally derived environmental Pseudomonas strains (env-Ps) for activity against a panel of 65 pathogens including Achromobacter spp., Burkholderia spp., Pseudomonas aeruginosa, and Stenotrophomonas spp. isolated from the lungs of cystic fibrosis (CF) patients. From more than 30,000 competitive interactions, 1,530 individual inhibitory events were observed. While strains from water habitats were not proportionate in antagonistic activity, MDR CF-derived pathogens (CF-Ps) were less susceptible to inhibition by env-Ps, suggesting that fewer natural products are effective against MDR strains. These results advocate for a directed strategy to identify unique drugs. To facilitate discovery of antibiotics against the most resistant pathogens, we developed a workflow in which phylogenetic and antagonistic data were merged to identify strains that inhibit MDR CF-Ps and subjected those env-Ps to transposon mutagenesis. Six different biosynthetic gene clusters (BGCs) were identified from four strains whose products inhibited pathogens including carbapenem-resistant P. aeruginosa. BGCs were rare in databases, suggesting the production of novel antibiotics. This strategy can be utilized to facilitate the discovery of needed antibiotics that are potentially active against the most drug-resistant pathogens.

**IMPORTANCE** Carbapenem-resistant P. aeruginosa is difficult to treat and has been deemed by the World Health Organization as a priority one pathogen for which antibiotics are most urgently needed. Although metagenomics and bioinformatic studies suggest that natural bacteria remain a source of novel compounds, the identification of genes and their products specific to activity against MDR pathogens remains problematic. Here, we examine water-derived pseudomonads and identify gene clusters whose compounds inhibit CF-derived MDR pathogens, including carbapenem-resistant P. aeruginosa.

## Abstract (precis)

Carbapenem-resistant P. aeruginosa is difficult to treat and has been deemed by the World Health Organization as a priority one pathogen for which antibiotics are most urgently needed. Although metagenomics and bioinformatic studies suggest that natural bacteria remain a source of novel compounds, the identification of genes and their products specific to activity against MDR pathogens remains problematic. Here, we examine water-derived pseudomonads and identify gene clusters whose compounds inhibit CF-derived MDR pathogens, including carbapenem-resistant P. aeruginosa.

## INTRODUCTION

The World Health Organization has identified 12 pathogens for which novel antibiotics are most urgently needed, and carbapenem-resistant Pseudomonas aeruginosa is considered a priority one threat (1). With 10 million deaths predicted annually by 2050 from multidrug-resistant (MDR) infections (2), the discovery of novel therapeutics effective against pathogens is needed. Biosynthetic gene clusters (BGCs) encode bacterial secondary metabolites whose function extends beyond the normal growth and metabolism of a cell by facilitating a variety of processes, including antibiotic production. Recent genomic studies suggest that environmental bacteria represent a continued source of novel BGCs that encode unique products (3–6). In particular, marine and freshwater-derived bacteria have been the most promising source of unique products. For example, the novel antibiotic nucleoside analogue 201A from the deep-sea marine actinomycete Marinactinospora thermotolerans SCSIO was recently discovered to inhibit MDR Staphylococcus aureus (7). Cocultures of marine invertebrates and associated bacteria led to the discovery of multiple antagonistic factors, including keyicin, an anthracycline that inhibits Gram-positive pathogens (8), in addition to β-carbolines and indolactam alkaloids that possess potent antimalarial activity (9). A natural product from the marine bacterium Pseudoalteromonas was able to inhibit a multidrug efflux pump, thus increasing the effects of antibiotics against MDR pathogens (10). Last, lake-derived pseudomonads were active at inhibiting Pseudomonas aeruginosa (11), as well as oomycete plant pathogens (12). Although the prediction of BGCs from metagenomics data and the discovery of new compounds suggest a continued source for antibiotic discovery, the abundance of novel compounds effective against MDR pathogens remains unknown.

Cystic fibrosis (CF) is an autosomal recessive genetic disease resulting from mutations in the cystic fibrosis transmembrane conductance regulator (*CFTR*) gene (13). Mutations within *CFTR* result in decreased chloride permeability within mucosal membrane tissue (14), leading to increased mucoid production in the respiratory track and ideal conditions for bacterial colonization and growth (15). CF patients are therefore susceptible to chronic pulmonary infections and are regularly colonized by species of Achromobacter (16), Stenotrophomonas (16), Burkholderia (17), and P. aeruginosa (18), all of which are known to have evolved drug resistance and present complications in the CF lung. Previously, we showed that CF-derived P. aeruginosa strains (CF-PAs) are susceptible to environmental *Pseudomonas* strains (env-Ps) which may also be a source of natural products that inhibit other pathogens, including MDR strains (11). env-Ps represent a large, genetically diverse group of bacteria (19, 20) that are ubiquitous in the environment and are readily isolated from soil (21, 22) and freshwater (23, 24) habitats. Moreover, env-Ps are known to produce a variety of secondary metabolites that are involved in functions ranging from plant health and disease to bioremediation and microbial inhibition (20). Because of the wide range in structure and function of these metabolites and our earlier findings that water-derived bacteria actively inhibit pathogens (11), we predicted that env-Ps from distinct water habitats would inhibit CF-derived pathogens (CF-Ps), including *Achromobacter* spp., *Burkholderia* spp., P. aeruginosa, and *Stenotrophomonas* spp.

To assay for antagonistic activity against pathogens, we investigated direct interactions between env-Ps and CF-Ps. A collection of pseudomonads was sampled across time and space, including strains from water systems in the United States, Hungary, and Germany. Using 471 env-Ps, we performed a population-level analysis in which these strains were genetically characterized using the *gyrB* gene and then tested for their ability to inhibit CF-Ps in one-on-one competitions. We identified several env-Ps with the ability to inhibit multiple CF-Ps. However, the ability to inhibit MDR CF-PAs was limited, suggesting that there are fewer novel natural products available for discovery against the most resistant pathogens. To target diverse loci whose products were active against the most devastating pathogens, env-P inhibition and phylogenetics were merged to identify genetically distinct antagonistic strains. Transposon (Tn) mutagenesis and genome sequencing were utilized on four env-Ps that inhibited multiple CF-Ps. Using this approach, we identified six dissimilar biosynthetic gene clusters (BGCs) whose products were able to inhibit MDR pathogens, including carbapenem-resistant P. aeruginosa. Computational analyses revealed that these BGCs differ from each other and were infrequent in the Joint Genome Institute (JGI) and NCBI databases, suggesting these strains may encode novel antagonistic factors. Although our results imply that fewer novel antibiotics are available for discovery against MDR pathogens, we offer a strategy to identify BGCs whose products are potentially active against pathogens.

## RESULTS

### Population-level diversity of environmental pseudomonads.

**FIG 1:**
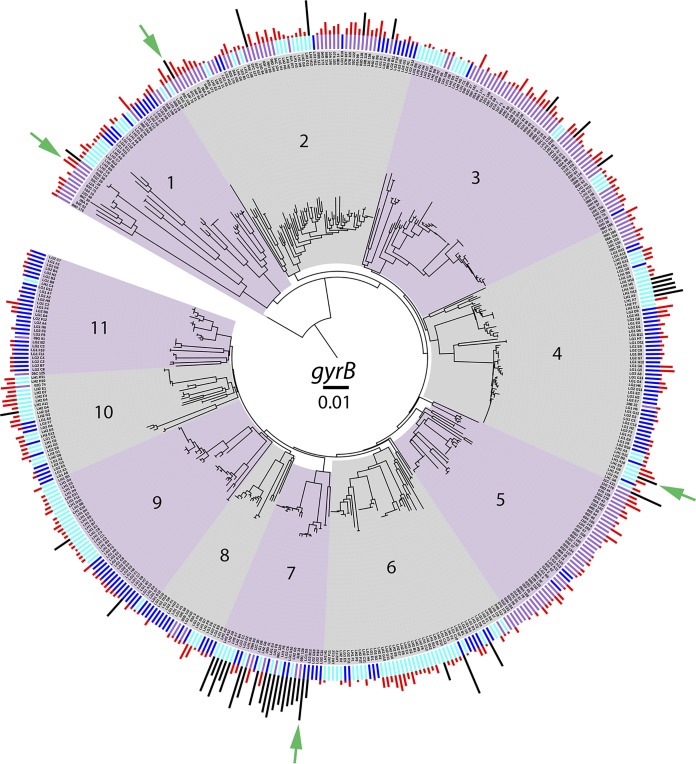
Phylogenetic analysis and antagonistic activity among env-Ps. Population structure for 471 environmental pseudomonads by neighbor-joining analysis of the *gyrB* sequence, merged with data for habitat (inner bars: purple, United States; dark blue, Germany; light blue, Hungary) and antagonistic activity (outer bars; black and red) against 65 CF-Ps. The magnitude of antagonism is indicated by bar height. Strains that inhibit more than nine pathogens are indicated by black bars. Populations are shaded and numbered 1 to 11. Tn mutagenesis was used to identify BGCs involved in antagonistic activity in strains 06C126, 09C129, LG1D9, and LH1G9 (indicated by the green arrows).

Strains were isolated from different water habitats to obtain a diverse group of env-Ps. Totals of 163, 160, and 148 strains were obtained from watercolumns in the United States, Germany, and Hungary, respectively, resulting in a collection of 471 pseudomonads. In the United States, Lake Erie has a surface area of 25,667 km^2^ and a mean depth of 19 m that borders Canada and has been impacted by human activity. During the time of sampling, the water temperature was 11.5°C, and the lake was not ice covered (25). Strains from Hungary were obtained from Lake Balaton, which is in the Transdanubian region and is the largest lake in central Europe; it has a surface area of 596 km^2^ and a mean depth of 3.2 m. During sampling, the lake was covered by thin ice (2 to 5 cm) with negligible snow cover, and the water temperature was 3.5°C (26). env-Ps from Germany were obtained from the Darss-Zingst estuary in the state of Mecklenburg-West Pomerania, which represents a brackish habitat and had a temperature of 4.0°C. The ecological factors within these habitats likely differ and may select for strains producing diverse metabolites within and between environments. As an initial examination of strain identity, the *gyrB* gene was amplified, sequenced, and BLAST searches were performed against the NCBI nucleotide database. All strains were identified as *Pseudomonas* at the genus level. A neighbor-joining phylogenetic tree was created from each of the 471 *gyrB* housekeeping gene sequences to visualize the population-level genetic diversity (Fig. 1). Eleven populations, each consisting of 18 or more strains, were identified based on nucleotide divergence and branching patterns. To investigate the ecological distribution of natural env-Ps by population, data corresponding to derived habitats were superimposed onto the phylogeny (Fig. 1, inner bars). All populations were observed to consist of isolates from at least two different habitats. Although few populations were composed of strains from one location, populations 3 and 5 contained strains mostly from the United States, and populations 11 and 6 were mostly from Germany and Hungary, respectively. Populations 4, 6, 9, and 10 consisted mostly of German and Hungarian samples, while populations 1, 2, 7, and 8 were mixed, suggesting that strains isolated from different habitats were closely related. While most strains differed at the *gyrB* locus, all populations had at least two strains with identical sequences, suggesting the presence of similar or clonal isolates. These results show the population-level genetic diversity among env-Ps and that the groups were composed of related strains from different water habitats.

### Environmental pseudomonads inhibit CF-derived pathogens.

**FIG 2:**
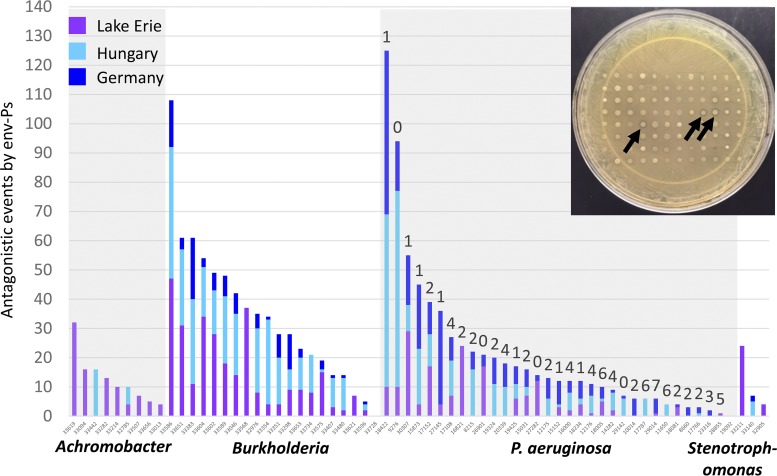
Antagonistic events of env-Ps against CF-Ps. A competition plate assay was used to determine antagonistic activity (inset). A total of 471 env-Ps were competed against 65 CF-Ps that resulted in 30,615 individual competitions. Of the 1,530 antagonistic events observed, activity originated from 579 U.S. (purple bars), 359 German (dark-blue bars), and 592 Hungarian (light-blue bars) env-Ps that inhibited the growth of nine *Achromobacter*, 19 *Burkholderia*, 31 P. aeruginosa, and three *Stenotrophomonas* strains. Black arrows indicate activity by three antagonistic strains. Only one *Burkholderia* strain and one P. aeruginosa strain were not inhibited by any env-P. P. aeruginosa strains were tested for susceptibility against eight antibiotics using the Kirby-Bauer disk assay. Numbers above the antagonistic bar data represent the number of drugs a particular pathogen resists, indicated in Table 1. Linear regression analysis showed that the number of antagonistic events to a pathogen was inversely related to antibiotic resistance.

With the evolution of pathogens that resist antibiotics, it is essential that new therapeutics are discovered to treat bacterial infections. We previously demonstrated that competitive interactions occur among pseudomonads and CF-PAs (11). Here, we sought to determine if environmental strains from water habitats were active in their ability to inhibit a panel of diverse CF-Ps. To assess competition, we utilized a plate-based assay in which strains were cocultivated in one-on-one competition and screened for antagonistic activity by a zone of clearing of at least 1 mm (Fig. 2, inset). We competed all 471 environmental isolates against a panel of 65 pathogens to determine if natural isolates could inhibit different CF-derived clinical strains. The collection consisted of nine *Achromobacter*, 20 *Burkholderia*, 33 P. aeruginosa, and three *Stenotrophomonas* strains. These pathogens are known to colonize the CF lung and are responsible for many of the bacterial infections caused in these patients (21, 27, 28). From all antagonistic assays, 1,530 inhibitory events were observed (see Table S1 in the supplemental material) that consisted of 579 events from the United States, 359 events from Germany, and 592 events from Hungary that directly inhibited the growth of nine *Achromobacter*, 19 *Burkholderia*, 31 P. aeruginosa, and three *Stenotrophomonas* strains (Fig. 2). Only one *Burkholderia* strain and one P. aeruginosa strain were not inhibited by any env-P. A chi-square test was used to determine whether the number of antagonistic events by env-Ps differed by location with respect to each of the four pathogen genera. The number of events was not proportional to strain isolation by location for *Achromobacter* (χ^2^ = 112.29, *df* = 2, *P* < 0.0001), *Burkholderia* (χ^2^ = 120.37, *df* = 2, *P* < 0.0001), *Pseudomonas* (χ^2^ = 12.69, *df* = 2, *P* < 0.0017), or *Stenotrophomonas* (χ^2^ = 23.61, *df* = 2, *P* < 0.0001). In general, env-Ps from the United States performed as well as or better than strains from the other two locations against CF-Ps, but strains from Germany and Hungary performed relatively poorly against *Achromobacter* and *Stenotrophomonas* pathogens. env-Ps from all locations performed similarly against CF-PAs.

We next determined if any of the env-Ps inhibited MDR CF-PAs. Using the Kirby-Bauer disk assay, we identified 11 of 33 CF-PAs that exhibited an MDR phenotype, defined as resisting the effects of four or more antibiotics (Table 1). In total, 104 antagonistic events were observed among 10 MDR CF-PAs (Fig. 2, numbers 4 to 7 above the bars). Of these pathogens, all 10 were antagonized by at least two env-Ps, and eight were inhibited by 10 or more env-Ps. Moreover, 73 antagonistic events were observed by 59 env-Ps against MDR CF-PA strains AU11650, AU12176, AU14282, AU17108, AU17787, AU18005, AU19092, AU28855, and AU29014 (Table S2), which are resistant to both carbapenems tested; this classified these clinical isolates as priority one pathogens, as defined by the WHO (1), for which antibiotics are urgently needed. CF-PA strain AU29014 is resistant to all known antibiotics except colistin and was only inhibited by six env-Ps. Since less env-P activity seemed to be observed against MDR CF-PAs (Fig. 2), a linear regression was performed to determine if the susceptibility of MDR pathogens was negatively related to the magnitude to which pathogens are resistant to antibiotics. The analysis showed that the number of antagonistic events dropped by nearly two with every unit increase in the number of antibiotics which a pathogen resists. Thus, the more-drug-resistant CF-PAs were the least antagonized by env-Ps. A similar trend was observed among CF-Ps and antagonistic activity observed from the env-Ps (Fig. 2); however, antibiotic susceptibility and resistance standards are not documented by the Clinical and Laboratory Standards Institute (22) for *Achromobacter*, *Burkholderia*, and *Stenotrophomonas* spp., so strains of these genera were not tested for an MDR phenotype. The results indicated that env-Ps engage in competitive interactions against CF-Ps, but significantly fewer antagonistic events were observed against MDR CF-PAs, suggesting that fewer natural drugs are available for discovery that are effective against the most-antibiotic-resistant pathogens.

**TABLE 1:** Antibiotic resistance of CF-PA strains

CF-PA strain	Antibiotic (concn [μg/disk]) resistance result^*a*^							
	CL 10	CB 100	C 30	MEM 10	IPM 10	NN 10	CIP 5	CAZ 30
AU8215	S	S	R	I	R	S	S	S
AU8660	S	R	R	S	S	S	S	S
AU9276	S	S	S	S	S	S	S	S
AU10014	S	R	I	S	S	S	S	R
AU11650^*b*^	S	R	R	R	R	S	R	R
AU12175	S	S	R	S	S	S	R	S
AU12176^*b*^	S	R	I	R	R	S	I	R
AU14282^*b*^	S	S	R	R	R	S	R	S
AU15031	S	S	R	S	S	R	I	S
AU15152	S	S	R	S	S	S	S	S
AU15873	S	S	R	S	S	S	S	S
AU16000^*b*^	S	R	R	S	R	S	R	S
AU16821	S	I	I	S	S	R	R	S
AU17108^*b*^	S	I	R	R	R	S	R	I
AU17152	S	S	R	S	S	S	R	S
AU17766	S	S	R	S	I	R	I	S
AU17787^*b*^	S	R	R	R	R	S	R	R
AU18005^*b*^	S	R	S	R	R	R	R	R
AU18081	S	S	R	S	R	I	I	S
AU18234	S	S	R	S	S	S	S	S
AU18422	S	S	S	S	S	S	S	R
AU19092^*b*^	S	R	R	R	R	S	S	I
AU19324	S	R	I	S	S	R	S	S
AU19425	S	S	R	S	S	S	I	S
AU20339^*b*^	S	S	R	I	R	R	R	S
AU23316	S	I	R	S	S	R	S	R
AU26901	I	S	I	S	S	R	I	S
AU27145	S	S	S	I	R	S	S	S
AU27282	S	S	I	S	S	I	S	S
AU28855^*b*^	S	R	R	R	R	R	I	S
AU29014^*b*^	S	R	R	R	R	R	R	R
AU29142	S	S	I	S	S	S	I	S
AU30307	S	S	S	S	S	S	R	S

a CL, colistin; CB, carbenicillin; C, chloramphenicol; MEM, meropenem; IPM, imipenem; NN, tobramycin; CIP, ciprofloxacin; CAZ, ceftazidime; S, susceptible; I, intermediate; R, resistant.

b MDR strains.

### Strategy to identify distinct env-P BGCs that inhibit pathogens.

Two environmental isolates from different populations, habitats, and with dissimilar antagonistic profiles were selected for Tn mutagenesis, the purpose being that these env-Ps likely produce distinct compounds, encoded by different BGCs, that inhibit CF-Ps. Strain 02C26 was isolated from the United States, mapped to population 1, and inhibited seven CF-Ps (Table S3); strain LH1G9 was from population 4, obtained from a freshwater lake in Hungary, and inhibited four P. aeruginosa and six *Burkholderia* strains. Although Tn insertion results identify a single mutated locus, secreted compounds may be encoded by multiple genes within a particular chromosomal region. If a BGC was disrupted by a Tn and the mutant exhibits a loss-of-inhibition (LOI) phenotype, that region was likely involved in antibiotic production and secretion. The wild-type strains were sequenced and annotated, and all had multiple predicted BGCs (Table S4).

**FIG 3:**
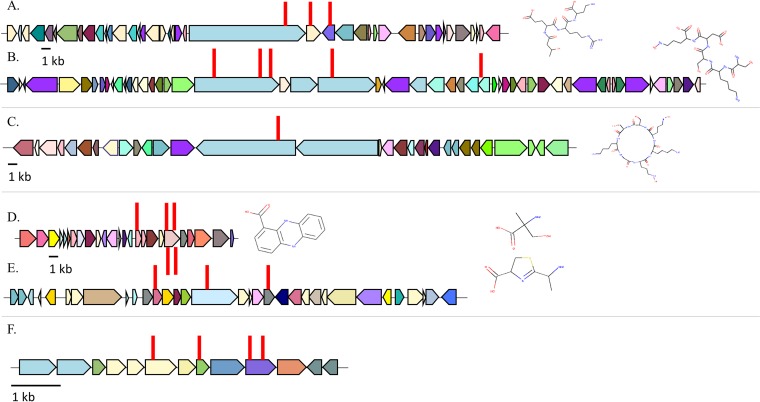
Tn insertions were identified in strains 06C126, LH1G9, 09C129, and LG1D9. (A to C) Tn-mutated BGCs with JGI ID numbers 161819466 (A) and 161819467 (B) were identified in strain 02C26, and BGC 161848994 (C) was identified in LH1G9. All three loci were predicted to encode a nonribosomal peptide. (D and E) In 09C129, BGCs 161816930 (D) and 161816936 (E) were identified and predicted to encode a phenazine and nonribosomal peptide, respectively. (F) With strain LG1D9, four Tn insertions were identified in genes that were not predicted to be a BGC. ORFs are represented by solid-color filled arrows; different colors represent dissimilar proteins (listed in Tables S4 to S9). Right and left pointed arrows signify loci on forward and reverse DNA strands, respectively. Red lines indicate the position of the Tn insertion. Predicted compound structures follow each BGC and were determined using PRISM.

**FIG 4:**
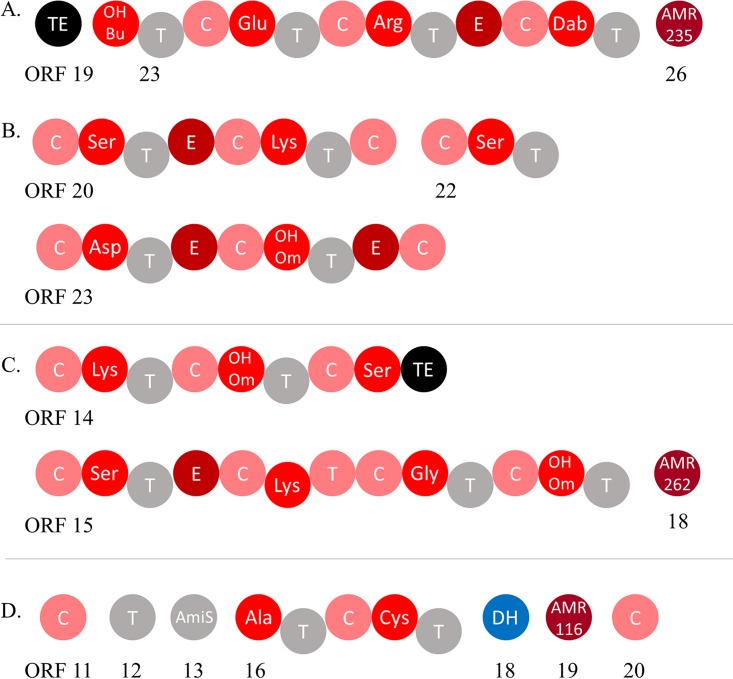
Predicted domains of NRPSs in env-Ps. (A and B) Strain 02C26 has two BGCs which were each predicted to encode NRPSs, as follows: ORF 23 was identified in BGC with JGI ID 161819466 (A) and ORFs 20, 22, and 23 were identified in BGC 161819467 (B). (C) Strain LH1G9 has two loci that encode an NRPS which were identified in ORFs 14 and 15 of BGC 161848994. (D) In strain 09C129, ORF 16 was found in BGC 161816936 and was predicted to encode an NRPS with associated ORFs 11 to 13 and 18 to 20. NRPS domains are shown as individual circles with predicted functions involved in condensation (C; in pink), adenylation (OHBu, 3-hydroxybutanoic acid; Dab, 2,4-diamino-butyric acid; and OHOm, *N*^5^-hydroxy-l-ornithine; in red), thiolation (T; in gray), epimerization (E; in dark red), and thioesterase (TE; in black). Other domains identified were antibiotic resistance (AMR235, MacB subunit of efflux pump; AMR262, MatE: efflux protein; and AMR116, puromycin major facilitator superfamily [MFS] transporter; in maroon), and dehydratase (DH; blue). Numbered ORFs correspond to Tables S4, S5, S6, and S8.

With both 02C26 and LH1G9 env-Ps, Tn insertions were found within BGCs (Table 2). For strain 02C26, eight independent LOI mutants were identified (Table 3). Three Tn insertions disrupted genes in a 53-kb BGC (JGI identifier [ID] 161819466 and Fig. 3A) that contained 41 predicted open reading frames (ORFs) (Table S5). ORFs 23 and 24 were disrupted and predicted to encode a nonribosomal peptide synthetase (NRPS) (Fig. 4A) and a hypothetical protein (Table 3), respectively. The other mutation occurred in ORF 26; ORFs 26 and 27 encode putative products of a macrolide antibiotic efflux system. The other five Tn insertions disrupted loci in a 79-kb gene cluster (JGI ID 161819467 [Table 2]) that contained 53 putative ORFs and was predicted to encode a bacteriocin-like compound (Fig. 3B and Table S6). Three Tn insertions were within ORF 20 (Table 3), which was predicted to encode an NRPS (Fig. 4B). The other two insertions occurred in ORFs 23 and 32, which were predicted to encode another NRPS and a zinc-dependent dipeptidase (Table 3), respectively. From all mutants, three NRPSs were identified within two BGCs (Fig. 4A and B) and likely contribute to the production of a nonribosomal peptide. In addition to these Tn-disrupted regions, other ORFs potentially encoding iron uptake systems were located within the BGC, suggesting that this locus may be associated with a siderophore (Table S6; ORFs 26, 42, and 47). Of the seven BGCs predicted in LH1G9, one mutant was identified that resulted in the LOI phenotype. The Tn insertion disrupted a BGC that was 64 kb (JGI ID 161848994 and Fig. 3C) and consisted of 30 ORFs (Table S7). ORF 14 was mutated (Table 3) and predicted to encode an NRPS (Fig. 4C). Based on the BGC size and gene content of the env-Ps 02C26 and LH1G9 (Fig. 3A to C), it is apparent that these loci are diverse in structure and likely encode distinct products that contribute to antagonistic activity.

**TABLE 2:** Tn-mutated BGCs among env-Ps 02C26, 09C129, LG1D9, and LH1G9

Strain	BGC size (kb)	Putative BGC product/function	BGC coordinates	JGI BGC ID no.	No. of Tn mutants
02C26	53	Pyoverdine/NRPS	1639452–1692405	161819466	3
	79	Pyoverdine/bacteriocin/NRPS	1906137–1985107	161819467	5
09C129	23	Phenazine	6197676–6220462	161816930	5
	50	NRPS	3874653–3924442	161816936	3
LG1D9^*a*^	6	Fatty acid metabolism	3420434–3426705		5
LH1G9	65	NRPS	2547071–2611595	161848994	1

a The Tn was not inserted into a BGC predicted by JGI IMG or antiSMASH.

**TABLE 3:** env-P-mutated genes involved in antagonistic activity

Strain	JGI BGC no.	ORF no.	No. of Tn inserts	Predicted function	JGI locus tag
02C26	161819466	23	1	NRPS	Ga0151585 111533
	161819466	24	1	Hypothetical	Ga0151585 111534
	161819466	26	1	Efflux pump	Ga0151585 111536
	161819467	20	3	NRPS	Ga0151585 111764
	161819467	23	1	NRPS	Ga0151585 111767
	161819467	32	1	Zinc-dependent dipeptidase	Ga0151585 111776
LH1G9	161848994	14	1	NRPS	Ga0199208 112386
09C129	161816930	16	1	Phenazine biosynthesis	Ga0139558 115541
	161816930	20	4	Phenazine biosynthesis	Ga0139558 115545
	161816936	11	1	NRPS	Ga0139558 113529
	161816936	16	1	NRPS	Ga0139558 113534
	161816936	20	1	NRPS	Ga0139558 113538
LG1D9		6	1	Flavin adenine dinucleotide binding protein	Ga0172616 112987
		8	1	Peroxiredoxin	Ga0172616 112989
		10	2	Fatty acid hydrolase	Ga0172616 112991

### Identification of BGCs whose products are potentially active against MDR CF-Ps.

We utilized the above-mentioned strategy to identify BGCs whose products inhibit multidrug- and carbapenem-resistant pathogens. Strain 09C129 was isolated from the United States, mapped to population 2 (Fig. 1), and inhibited 10 CF-Ps (Table S3), of which AU12176 and AU17108 were MDR CF-PAs (Table 1). Strain LG1D9 was isolated from brackish water in Germany, mapped to population 11, and antagonized 20 CF-Ps, including the growth of CF-PAs AU12176, AU17108, and AU20339 (Table S3); strains AU12176 and AU17108 were resistant to four and five antibiotics, respectively, including both carbapenems, while AU20339 was resistant to four antibiotics, including imipenem, with an intermediate phenotype to meropenem (Table 1). Strain LG1D9 also inhibited 11 other P. aeruginosa strains and seven *Burkholderia* strains. Utilizing the phylogenetic, habitat, and antagonistic data, env-P 09C129 and LG1D9 data were unique compared to each other and to 02C26 and LH1G9, suggesting that the BGCs would be distinct.

For strain 09C129, two BGCs were identified from eight independent Tn insertions (Table 2). Five Tn insertions were found in a BGC that was 23 kb (Fig. 3D; JGI ID 161816930) and was predicted to encode a phenazine. Within this cluster (Table S8), ORFs 16 and 20 had one and four Tn insertions and encode a putative phenazine biosynthesis protein A/B and a phenazine biosynthesis protein PhzE, respectively (Table 3). Phenazines are nitrogen-containing heterocyclic compounds that are known to have broad-spectrum antibiotic properties and involvement in virulence (23). Additionally, multiple genes were predicted to encode a secretion system. The other insertions occurred in three different loci in a 50-kb BGC (Fig. 3E; JGI ID 161816936) that has 32 predicted ORFs (Table S9). One mutant had the Tn insertion within ORF 11 (Table 3), which was predicted to encode a condensation domain-containing protein (Fig. 4D). The other mutants had Tn insertions in ORFs 16 and 20 (Table 3), which were predicted to encode amino acid adenylation and condensation domain proteins (Fig. 4D), respectively, all of which are characteristic domains of an NRPS. These results suggest that these two BGCs play an important role in producing a nonribosomal peptide that actively inhibits MDR CF-PAs. For strain LG1D9, four LOI mutants were identified, and the Tn insertions were found within a 6.2-kb region (Table S10), disrupting three potential genes (Fig. 3F). ORF 6 was predicted to encode a flavin adenine dinucleotide (FAD) binding protein, ORF 8 was predicted to encode a peroxiredoxin, and two Tn insertions were identified in ORF 10, which was predicted to encode a fatty acid hydrolase (Table 3). This locus was not a predicted BGC by JGI GOLD (24), antiSMASH (29), or PRISM (30).

### Frequency of identified BGCs among sequenced genomes.

Two databases were utilized to determine if the discovered BGCs were unique among sequenced genomes. First, BLAST searches of each BGC were performed against the NCBI nucleotide Microbial Genomes database to search for other similar gene clusters. Although similar BGCs were identified in NCBI, none were 100% identical to other gene clusters or frequent in the database (Table S11). For strain 02C26, the 53-kb gene cluster (JGI ID 161819466) had a query cover of 95% and 82% nucleotide identity to a locus in Pseudomonas putida KT2440 (31); the next closest hit was Pseudomonas monteilii strain USDA-ARS-USMARC-56711, which showed query coverages of 59% and 80% nucleotide identity. The other BGC in strain 02C26 (JGI ID 161819467) was most similar to Pseudomonas plecoglossicida strain XSDHY-P, with query coverages of 28% and 85% nucleotide identity. Interestingly, the closest hits of each BGCs were identified in different *Pseudomonas* species. For strain 09C129, which was able to inhibit MDR CF-PAs, both BGCs were identified among Pseudomonas chlororaphis strains. One BGC (JGI ID 161816930) was almost identical to a gene cluster in strain DSM 50083; the other BGC (JGI ID 161816936) had a coverage of 69%, suggesting that the loci were different in content. We identified one BGC in the other two strains, LG1D9 and LH1G9, and each of those clusters was closely related to loci in only two other strains from the species Pseudomonas fluorescens and Pseudomonas veronii, respectively. Thus, the BGCs were rare in the NCBI Microbial Genomes database.

**FIG 5:**
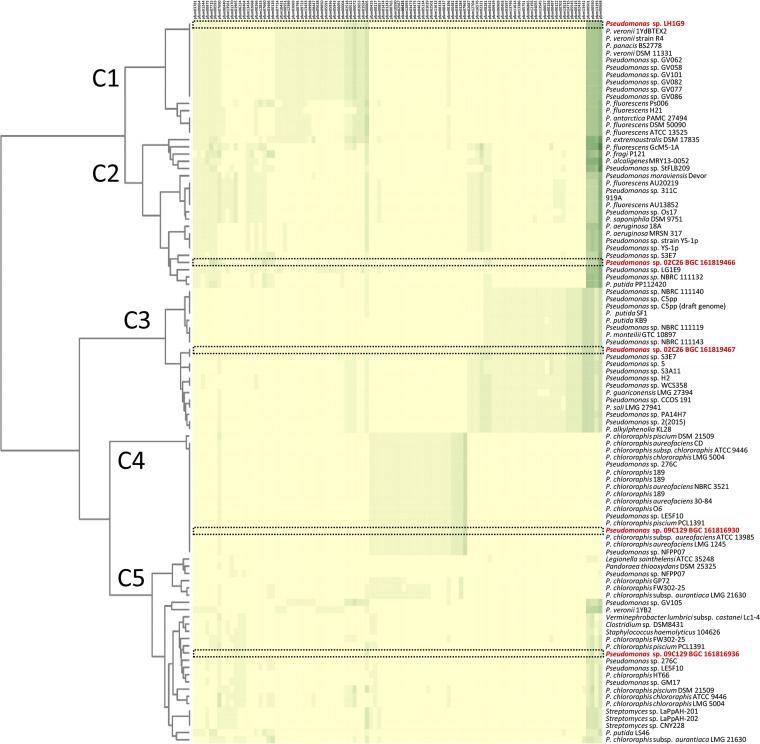
Heat map of similar Pfam protein families in the JGI ABC to 02C26, LH1G9, 09C129, and LG1D9 BGCs. The 95 most similar BGCs were identified and used to show similarities and differences within each cluster. Five clades were generated in the analysis, C1 to C5. The JGI ABC Pfam designations are listed on the top *x* axis and correspond to Table S11. The heat map color shading represents the number of each Pfam protein family in a BGC. Unique, single, and multiple protein families in a BGC range from 0 to 5, with yellow representing 0 protein families and lightest green to darkest green representing 1 to 5 protein families. The left *y* axis consists of BGC phylogeny determined by number and content of similar protein families. Tree branch lengths were determined using the Jaccard index scores of all protein families. The right *y* axis lists strains, with 02C26, LH1G9, and 09C129 in red; their BGC is boxed with a black dashed line.

Because nucleotide divergence within a BGC may be greater than the amino acid similarity, env-P BGCs were also analyzed using the JGI Atlas of Biosynthetic Gene Clusters (ABC) and grouped based on Pfam (32). To date, the JGI database has nearly 100,000 sequenced bacterial genomes and over 1.1 million predicted BGCs. To visualize BGC diversity, a heat map was generated using the top 19 BGCs identified in the JGI-ABC from 02C26, 09C129, and LH1G9, giving hits to the most related 95 loci based on Pfam content (Fig. 5). The results showed that each BGC was diverse and grouped into clades C1 to C5, suggesting dissimilar protein content (Fig. S1). The BGC from LH1G9 grouped with 16 other strains in C1, and 10 of those were identical. Although they are the same based on Pfam content, these 11 BGCs represent less than 0.0009% of all loci in the database, suggesting that the clusters were rare. The BGCs in strain 02C26, JGI IDs 161819466 and 161819467, grouped within C3 and C2, respectively. Clade C2 consists of 21 strains, and no loci exactly matched the BGC; C3 consists of 20 strains, and only *Pseudomonas* sp. strain S3E7 has an identical BGC. Strain 09C129 has BGCs JGI IDs 161816930 and 161816936 that grouped within C4 and C5. C4 consisted of 17 strains, including BGC 161816930, which were identical. As with LH1G9, these identical loci represent only a small percentage of the BGCs in the database. Clade C5 consists of 26 strains, and no BGC was identical to BGC JGI ID 161816936. Most strains within all groupings consisted of pseudomonads. LG1D9 was not used in this analysis since the Tn-mutated loci were not within a BGC identified by antiSMASH or the JGI-ABC. Together, these results suggest that the env-P BGCs were distinct from each other and infrequent among bacteria in the JGI-ABC database.

## DISCUSSION

Recent studies suggest that water-derived bacteria encode a collection of undiscovered natural products that might be devolved to target pathogens (4, 8, 33, 34). Given that env-Ps thrive in freshwater and have known applications in agriculture, bioremediation, and health (5, 20), we reasoned that pseudomonads isolated from different aquatic systems could maintain unique metabolic pathways and produce compounds that inhibit pathogens. With over 1,500 antagonistic events observed, water-derived env-Ps represent a source of strains that actively inhibit CF-derived clinical strains (Fig. 2). Moreover, the antagonistic plate assay used in this study only tested for activity under one medium type. Changing nutrients may affect the regulation of other metabolites involved in activity. Thus, the results presented here likely represent a conservative estimate of activity by these strains and possibly the production of novel drugs. Even in the current antibiotic crisis, uncertainty remains as to whether the number of undiscovered drugs that are effective against MDR pathogens are many or few. Our data show that env-Ps were less active against MDR pathogens, which implies that fewer drugs may be available for discovery that inhibit MDR pathogens. Although these results only represent activity from culturable env-Ps, we propose that this prediction extends to unculturable strains since certain genes, acquired through lateral transfer, confer resistance against multiple antimicrobial compounds regardless of cultivation. For instance, the NDM-1 gene encodes an enzyme that confers resistance to most β-lactams (35, 36), multidrug efflux pumps recognize different antibiotics so one pump is effective against different drugs (37–39), and plasmids carry resistance to “last-resort antibiotics” (40), including carbapenem and colistin (41), which are readily acquired through conjugation. Because a single mechanism can confer resistance against multiple antibiotics, it is unlikely that novel effective compounds that evade resistance mechanisms are abundant. The reduced activity against MDR CF-PAs that we observe supports the idea that few natural products are available for discovery that inhibit the growth of MDR pathogens.

With the increased interest in natural products as a source for combatants that inhibit pathogens, powerful computational programs, such as antiSMASH (42), ClusterFinder (4), and MIBiG (43), were developed and have identified thousands of BGCs from environmental strains; however, connecting gene clusters to novel antibiotic discovery is difficult. To facilitate this process, we merged population-level structure (Fig. 1), antagonistic events (Fig. 2), and Tn mutagenesis to identify different loci whose products are involved in antagonistic activity (Fig. 3). PRISM was used to identify putative structures based on gene content, and the results suggested the production of dissimilar compounds (Fig. 3A to E). The two BGCs identified in strain 02C26 were genetically distinct and separated by 213.7 kb on the chromosome. The 53-kb BGC (Fig. 3A) was predicted to encode an NRPS (Fig. 4A). Adenylation, condensation, and thiolation domains are characteristic of these structures and likely contribute to the production of a nonribosomal peptide. Moreover, NRPSs are diverse in structure and function and synthesize a wide variety of peptides (4, 44), including most known antibiotics (45). Other genes within the BGC may be involved in modifying the peptide or in export of the compound. For instance, ORFs 26 and 27 (Table S5) have putative products predictive of macrolide efflux systems. Macrolides are a class of antibiotics, suggesting that the NRPS may encode a macrolide-like compound and efflux system for drug production and resistance. This gene cluster was similar to a locus in P. putida KT2440; although this strain has been well characterized and recently resequenced to further understand its metabolic capabilities (31, 46), it has not previously been shown to inhibit pathogens. The other identified BGC in 02C26 was 79 kb (Fig. 3B and Table S6), consisting of 53 putative ORFs, and was predicted by antiSMASH to encode two large NRPSs and a small NRPS (Fig. 4B) that showed similarity to components involved in the synthesis of pyoverdine, a fluorescent siderophore produced by certain pseudomonads (47, 48). Some bacteria have been shown to produce sideromycins that are siderophores with an attached antibiotic (49). These “Trojan horse” molecules inhibit bacteria that sequester iron from the environment. Although sideromycins have not been identified in *Pseudomonas* spp., it is likely that env-Ps express such compounds, especially since *Pseudomonas* spp. as a group are known for diverse siderophore production. To further elucidate the functions of these loci, a gene-by-gene knockout deletion study may shed light upon the encoded product and demonstrate if one locus depends on the other for activity but not the reciprocal.

In strain 09C129, two BGCs were also identified and predicted to encode a phenazine and NRPS, which were 23 kb (Fig. 3D and Table S8) and 50 kb (Fig. 3E and Table S9) in size, respectively. Phenazines are characterized by a nitrogen-containing heterocyclic compound and are decorated by different functional groups that contribute to their structural and metabolic diversity (50). These compounds have a wide range of activity that contributes to their behavior and ecological fitness (23), and they have known antibiotic activity among *Pseudomonas* strains (51–53). In addition to phenazine biosynthesis genes, the BGC has 10 loci that were predicted to encode a secretion system. The other BGC was predicted to encode an NRPS (Fig. 4D). ORF 16 encodes the characteristic adenylation, thiolation, and condensation domains, while ORFs 11 to 13, 19, and 20 were also predicted to contribute to the production of the peptide (Fig. 4D). This collection of domains, although not within a single NRPS, may reflect recombination events between different domains with the potential to synthesize a hybrid NRPS, a phenomenon that has been observed in P. aeruginosa (54). To that end, the most closely related genes that encode the adenylation and condensation domains are from Bacillus (Table S9, ORF11) and *Pseudomonas* (Table S9, ORF16), suggesting that horizontal gene transfer contributed to the evolution of this locus.

In 02C26 and 09C129, both BGCs in each strain were predicted to contribute to the production of the antagonistic factor since transposon insertions in these loci produced a LOI phenotype. Coregulation of metabolites from distinct gene clusters has been identified in other organisms. Pseudomonas protegens Pf-5 produces two antibiotics, 2,4-diacetylphloroglucinol (DAPG) and pyoluteorin, whose BGCs are 3.7 Mb apart (55). An intermediate of DAPG not only activates the genes which produce pyoluteorin in strain Pf-5, but it also signals other P. protegens strains to produce pyoluteorin (56). This type of cooperative regulation among organisms has also been observed among fungi (57). Penicillium fuscum and Penicillium camemberti*/*P. clavigerum are extremophiles that, when cultured together, produce berkeleylactone A, an antibiotic that inhibits methicillin-resistant Staphylococcus aureus, Streptococcus pyogenes, and Bacillus anthracis. Although the mechanism of regulation is unknown, both species must be present to produce the drug, which suggests that different gene clusters are involved in compound synthesis. Only one BGC that contributed to antagonistic activity was identified between strains LH1G9 (Fig. 3C) and LG1D9 (Fig. 3F). The gene cluster in LH1G9 was predicted to encode two NRPSs (Fig. 4C). Although only one NRPS was identified with Tn mutagenesis, both of these loci might be involved in the production of a single peptide, considering their close proximity in the genome. It is also possible that other substances, metabolites, and regulators could play a synergic role for the observed effects that were not identified by the Tn screen. Furthermore, antagonistic activity by prophage may occur through lysis of the phage itself (58); however, no Tn inserts that disrupted a prophage were identified in this study.

A goal of this study was to identify novel BGCs that can be further pursued to identify and inform the production of new, potentially active antagonistic compounds. To accomplish this task, BGCs were mined in the NCBI and JGI ABC databases. Both searches showed that the BGCs were rare in the databases, and the heat map provided a visual representation of similar gene clusters (Fig. 5). No BGCs, including ones from the same strains, grouped into the same clade, which reflects the diversity of the loci. Although only the top 95 BGCs were searched in the JGI ABC, the results suggest that pseudomonads have clusters that are similar to well-known antibiotic producers (Fig. 5). For instance, three Streptomyces strains grouped in C5, proving evidence that the antagonistic *Pseudomonas* strains encode potentially active products that may represent an alternative source of effective compounds. The identification of BGC gene content through Tn mutagenesis (Fig. 3) and its predicted structure (Fig. 4) together with mining of those loci in databases (Fig. 5), the approach we adopted, can provide valuable information on the products and assist in the identification of new compounds. In addition, the wild-type and mutant strains can be biochemically coanalyzed to pinpoint active fractions through techniques such as high-performance liquid chromatography with subsequent purification and characterization of the active compounds. This strategy can facilitate the discovery of new compounds with potential for therapeutic use. env-Ps 09C129 and LG1D9 represent exceptional candidates for this workflow because both inhibited carbapenem-resistant CF-PAs that are priority one pathogens for which new antibiotics are currently needed (1). Finally, this drug discovery approach was proven effective in the Tiny Earth and Small World Initiative teaching curricula (59). Thus, the strategy used here could be implemented worldwide in a student crowdsourcing effort, in parallel with the Tiny Earth Chemistry Hub, to hasten the discovery of rare products to be used for the development of antibiotics against highly resistant pathogens.

## MATERIALS AND METHODS

### Strain isolation and growth conditions.

Water samples were obtained from Lake Erie in the United States in February 2012, from the shore of the Darss-Zingst estuary in Germany in February 2016, and from Lake Balaton off the shore of Tihany in Hungary in February 2016. The Lake Erie was sampled during surveys aboard the Canadian Coast Guard Ship (CCGS) *Griffon*. U.S. water samples were obtained from the Central Basin station EC1326 and collected from a depth of 1 m and a temperature of 1.5°C using a 10-liter Niskin bottle on a metered winch. For German and Hungarian samples, surface water was obtained from shore using a 1-liter Niskin bottle. All water samples were passed over 0.2-μm-pore-size 47-mm single-wrapped filters (Pall Corporation) to capture bacteria. Filters were then cultured on cetrimide agar (Fluka Analytical) at 23°C to select for pseudomonads. To purify strains, colonies were picked and restreaked two times on sterile nutrient broth (NB) solid medium (BD Difco) with 1.5% agar (BD Difco). CF-Ps, including P. aeruginosa, and different species of *Achromobacter*, *Burkholderia*, and *Stenotrophomonas* were gifted by John LiPuma at the University of Michigan. env-Ps and CF-Ps were grown at 23°C and 37°C, respectively, in NB liquid or agar medium. For transposon (Tn) mutagenesis (described below), the *Pseudomonas* strain was grown in NB. The Escherichia coli helper strain HB101 was grown in lysogeny broth (LB) liquid medium with 150 μg/ml ampicillin (Ap), and strain CC118 carrying pBAM1 was grown in LB with 50 μg/ml kanamycin (Km) and 30 μg/ml chloramphenicol (Cm), as previously described (60). E. coli strains were incubated at 37°C.

### Gene sequencing and phylogenetic analysis.

For gene sequencing, bacterial strains were grown in liquid culture for 2 days in NB at 23°C with shaking. A 10-μl sample was treated with Lyse-N-Go (Thermo Fisher Scientific, Rockford, IL) to extract and prepare genomic DNA as the template for PCR. Primers targeting the *gyrB* gene (*gyrB* 271 forward primer, 5′-TCB GCR GCV GAR GTS ATC ATG AC-3′; *gyrB* 1022 reverse primer, 5′-TTG TCY TTG GTC TGS GAG CTG AA-3′) were used to amplify and sequence the locus. PCR conditions were 92°C denaturation for 10 s, 65°C annealing for 60 s, and elongation at 72°C for 90 s, repeated 29 times. One*Taq* DNA polymerase (New England BioLabs) was used for amplification. Sanger sequencing was performed by the University of Chicago Comprehensive Cancer Center DNA Sequencing and Genotyping Facility. A nucleotide alignment was generated from 652 bp of the *gyrB* gene, and a neighbor-joining tree was constructed using Jukes-Cantor nucleotide distance measurement in CLC Main Workbench. Bootstrapping was performed in 100 replicates. The iTOL program was used to view the tree and overlay data corresponding to antagonistic activity (61).

### Antagonistic activity.

env-Ps were cultured for 20 h in NB medium at 23°C with shaking in a 96-well plate prior to the assay. To generate a bacterial lawn of the pathogen, 50 μl of a single culture was spread on NB agar plates. Subsequently, 1 μl of each environmental strain was transferred to the lawn using a 96-pin replicator (Boekel microplate replicator). Strains were cocultured at 23°C for 20 h to allow growth of the environmental strains. Assays were then temperature shifted to 37°C to provide for optimal growth of the pathogen. Antagonistic activity was scored as positive for a given Env-P if a zone of clearing of at least 1 mm was produced in the pathogen lawn surrounding the env-P strain. To confirm positive results, all inhibitory strains were selected and replicated at least three times against all pathogens. A chi-square test was used to determine whether the number of events differed by location with respect to each of the four pathogen genera. The expected proportions of the total observed events were 0.34, 0.31, and 0.35 for strains from Germany, Hungary, and the United States, respectively, because isolated strains from these countries represented 34%, 31%, and 35% of the 471 tested strains, respectively, each of which was tested against all pathogens in each genus. Linear regression was used to determine whether the number of observed events was related to the known antibiotic resistance of *Pseudomonas* pathogens. The model included the number of strains derived from each location for which an event was observed as the response variable, with the location and the number of antibiotics (0 to 8) to which a pathogen is known to be resistant as predictors. The interaction between predictors was also included in the model to determine whether environmental isolates respond differently to the resistance of pathogens (62).

### Antibiotic susceptibility test.

The Kirby-Bauer disk diffusion susceptibility assay was used to test for antibiotic resistance (63). P. aeruginosa pathogens were spread plated onto Mueller-Hinton agar (BD, Difco) medium from an overnight culture using a sterile cotton swab. The following eight antibiotic disks (BD BBL) were placed over the spread plated strains: colistin (10 μg), carbenicillin (100 μg), chloramphenicol (30 μg), meropenem (10 μg), imipenem (10 μg), tobramycin (10 μg), ciprofloxacin (5 μg), and ceftazidime (30 μg). The diameters of the zones of inhibition were measured after 20 h of incubation. Pathogens were considered resistant if the zones of inhibition were less than 8 mm for colistin, 13 mm for carbenicillin, 12 mm for chloramphenicol, 13 mm for meropenem, 13 mm for imipenem, 12 mm for tobramycin, 15 mm for ciprofloxacin, and 14 mm for ceftazidime (BD BBL Sensi-Disc antimicrobial susceptibility test disks).

### Genome sequencing of isolated env-P strains 02C26, 09C129, LG1D9, and LH1G9.

Genomic DNA was extracted using the Wizard Genomic DNA purification kit (Promega). PacBio sequencing was performed by the University of Delaware DNA Sequencing and Genotyping Center. Genomic DNA was sheared using g-TUBE to 20-kb fragments (Covaris). The PacBio libraries were prepared using the standard PacBio protocol for 20-kb libraries (20-kb template preparation using the BluePippin size selection system). Each sample library was sequenced on PacBio RS II instrument with one single-molecule real-time (SMRT) cell using P6-C4 chemistry with a 6-h movie. The genome was assembled using PacBio Hierarchical Genome Assembly Process 3. The reads of the inserts were filtered by a quality score of 0.8 and read length of 1 kb (64). All assemblies folded into one contig.

### Transposon mutagenesis.

Triparental mating was used to deliver the Tn*5* mini-transposon from pBAM1 in E. coli strain CC118 with helper strain HB101 to *Pseudomonas* strains 02C26, 09C129, LG1D9, and LH1G9 (60). E. coli and *Pseudomonas* strains were cultured overnight, as described above. One milliliter of cells was washed with 10 mM MgSO_4_ to remove any traces of antibiotics. One hundred microliters of each strain (CC118, HB101, and one env-P strain) was mixed together in a 1:1:1 ratio and centrifuged. The pelleted cells were resuspended in 10 μl of 10 mM MgSO_4_, spotted on NB, and incubated at 30°C for 24 h. The cells were scraped from the plate and resuspended in 200 μl of 10 mM MgSO_4_, and 100 μl was plated onto solid cetrimide agar with 50 μg/ml to select for *Pseudomonas* transconjugants. Transconjugants were replica plated onto a sensitive P. aeruginosa pathogen and screened for mutants exhibiting a loss-of-antagonism phenotype. Mutant screens for 02C26, 09C129, LG1D9, and LH1G9 were performed on the sensitive pathogens AU10014 (P. aeruginosa), AU17108 (P. aeruginosa), AU33589 (Burkholderia cenocepacia GIIIb), and AU33586 (Burkholderia cepacia), respectively.

### Mutant DNA extraction and ARB PCR.

Genomic DNA was extracted from the mutant strains 02C26, 09C129, LG1D9, and LH1G9 using the Wizard Genomic DNA purification kit (Promega). Arbitrary PCR (ARB-PCR) was used to amplify the genomic DNA flanking the Tn insert (60, 65). Two PCR cycles were performed. ARB-PCR I was performed using 2 μl of genomic DNA and 5 μM primer ARB6 (GGCACGCGTCGACTAGTACNNNNNNNNNNACGCC), in combination with 5 μmol/liter primer ME-I-extR (CTCGTTTCACGCTGAATATGGCTC) or 5 μmol/liter primer ME-O-extF (CGGTTTACAAGCATAACTAGTGCGGC). The conditions for the ARB-PCR I were 5 min at 95°C, six cycles of 30 s at 95°C, 30 s at 30°C, and 90 s at 72°C, 30 cycles of 30 s at 95°C, 30 s at 45°C, and 90 s at 72°C, and an extension period of 4 min at 72°C. For the second round of ARB-PCR, 1 μl of ARB-PCR I product was used as the template. ARB-PCR II was performed using 1 μl of ARB-PCR I product and 5 μmol/liter primer ARB2 (GGCACGCGTCGACTAGTAC) in combination with 5 μmol/liter primer ME-I-intR (CAGTTTTATTGTTCATGATGATATA) or 5 μmol/liter primer ME-O-intF (AGAGGATCCCCGGGTACCGAGCTCG). The conditions for ARB-PCR II were 60 s at 95°C, 30 cycles of 30 s at 95°C, 30 s at 52°C, and 90 s at 72°C, followed by an extension period of 4 min at 72°C. PCR purification was performed on each ARB-PCR II product using the NucleoSpin gel and PCR cleanup kit (Macherey-Nagel). Samples were sequenced at the University of Chicago Comprehensive Cancer Center DNA Sequencing and Genotyping Facility using either ME-I intR primer or ME-O intF primer.

### Genome annotation and BGC characterization.

Genomes were annotated using the JGI Genomes On Line Database (GOLD [24, 66]). BGCs were initially identifed by utilizing both the JGI Integrated Microbial Genomes (IMG) system (67, 68) and antibiotics & Secondary Metabolite Analysis Shell (antiSMASH [42, 69]). JGI IMG provides the number of BGCs present in a genome under the Genome Statistics section, and antiSMASH is a program dedicated to the identification of gene clusters that encode secondary metabolites. BGCs were further characterized through the JGI IMG Atlas of Biosynthetic gene Clusters (ABC [32]). A heat map was constructed using the JGI ABC portal to visualize BGC diversity. BGCs were searched in the database for protein families (Pfam) that had similar content based on the number and type of protein families. The top 95 hits were used to generate the heat map. The Jaccard index and modified Jaccard index scores were applied to determine similar clusters.

### Data availability.

All *gyrB* gene sequences are available through GenBank accession numbers MH671920 to MH672390. The genomes of strains 02C26 (LE5C2), 09C129 (LE6C9), LG1D9, and LH1G9 are available through GenBank accession numbers CP025262, CP025261, CP026881, and CP026880 and JGI IMG genome ID numbers 2706794715, 2703719185, 2716884900, and 2740891816, respectively.

## Supplementary Material

Supplemental file 1
