# Supplementary material for: Loci Encoding Compounds Potentially Active against Drug-Resistant Pathogens amidst a Decreasing Pool of Novel Antibiotics
Source: Appl Environ Microbiol. 2019 Nov 14;85(23):e01438-19. doi: 10.1128/AEM.01438-19 (PMC6856318; doi:10.1128/AEM.01438-19)
Supplement: Supplemental file 1 [file AEM.01438-19-s0001.pdf]

Table S1. Env-P antagonistic activity against pathogens.

		Env-P activity		
Strain #	CF-P	Germany	Hungary	Lake Erie
AU33507	*Achromobacter dolens*	0	0	7
AU33094	*A. dolens*	0	0	16
AU33214	*A. dolens*	0	0	10
AU33619	*A. insuavis*	0	0	32
AU33013	*A. ruhlandii*	0	0	4
AU32785	*A. xylosoxidans*	0	6	4
AU33282	*A. xylosoxidans*	0	0	13
AU33442	*A. xylosoxidans*	0	16	0
AU33656	*A. xylosoxidans*	0	0	5
AU33383	*Burkholderia diffusa*	21	29	11
AU32976	*B. cenocepacia GIIIa*	5	22	8
AU33046	*B. cenocepacia GIIIa*	7	21	14
AU33596	*B. cenocepacia GIIIa*	1	2	2
AU33602	*B. cenocepacia GIIIa*	6	15	28
AU33407	*B. cenocepacia GIIIb*	1	10	3
AU33480	*B. cenocepacia GIIIb*	1	11	2
AU33589	*B. cenocepacia GIIIb*	7	23	18
AU33586	*B. cepacia*	16	45	47
AU33604	*B. cepacia*	3	17	34
AU33568	*B. contaminans*	0	0	37

AU33575	*B. multivorans*	3	1	15
AU33621	*B. multivorans*	0	0	7
AU33651	*B. multivorans*	4	26	31
AU33728	*B. multivorans*	0	0	0
AU33734	*B. multivorans*	0	13	8
AU33653	*B. seminalis*	3	11	9
AU33298	*B. vietnamiensis*	12	7	9
AU33351	*B. vietnamiensis*	8	16	4
AU33354	*B. vietnamiensis*	1	29	4
AU8215	*P. aeruginosa*	6	16	0
AU8660	*P. aeruginosa*	3	0	0
AU9276	*P. aeruginosa*	17	67	10
AU10014	*P. aeruginosa*	6	0	0
AU11650	*P. aeruginosa*	0	4	0
AU12175	*P. aeruginosa*	7	6	0
AU12176	*P. aeruginosa*	4	6	1
AU14282	*P. aeruginosa*	1	6	2
AU15031	*P. aeruginosa*	6	3	7
AU15152	*P. aeruginosa*	8	1	3
AU15873	*P. aeruginosa*	22	19	4
AU16000	*P. aeruginosa*	4	6	2
AU16821	*P. aeruginosa*	0	0	24
AU17108	*P. aeruginosa*	8	12	7

AU17152	*P. aeruginosa*	11	11	17
AU17766	*P. aeruginosa*	2	1	0
AU17787	*P. aeruginosa*	0	6	0
AU18005	*P. aeruginosa*	4	1	5
AU18081	*P. aeruginosa*	1	0	3
AU18234	*P. aeruginosa*	6	2	4
AU18422	*P. aeruginosa*	56	59	10
AU19092	*P. aeruginosa*	0	0	0
AU19324	*P. aeruginosa*	9	11	0
AU19425	*P. aeruginosa*	6	5	6
AU20339	*P. aeruginosa*	8	10	0
AU23316	*P. aeruginosa*	2	0	0
AU26901	*P. aeruginosa*	4	0	17
AU27145	*P. aeruginosa*	32	0	4
AU27282	*P. aeruginosa*	2	0	12
AU28855	*P. aeruginosa*	0	0	1
AU29014	*P. aeruginosa*	5	0	1
AU29142	*P. aeruginosa*	1	6	0
AU30307	*P. aeruginosa*	17	9	29
AU33140	*S. maltophilia*	2	5	0
AU32905	*S. maltophilia*	0	0	4
AU33211	*S. maltophilia*	0	0	24
	Totals by Habitat	359	592	579

Total	1530

Table S2. Env-Ps antagonistic activity against MDR CF-PAs.

	MDR CF-PAs						
Env-Ps	AU12176	AU14282	AU17108	AU11650	AU17787	AU18005	AU29014
LG1B6			+				
LG1C10	+	+	+			+	
LG1D1	+						
LG1D8			+			+	
LG1D9	+		+				
LG1D10			+				
LG1E4			+				
LG1E9	+		+				
LG1F4			+				
LG1G5						+	
LG1G6						+	
LG2C6							+
LG2C9							+
LG2D10							+
LG2E3							+
LG2E8							+
LH1A4					+		
LH1A5	+		+				
LH1A6					+		
LH1A7	+						

LH1A10		+		+		
LH1B5					+	
LH1B7		+			+	
LH1C9					+	
LH1D6	+					
LH1D7	+					
LH1D11	+					
LH1E6		+				
LH1E9				+		
LH1F4				+		
LH1F6	+		+			+
LH1F12					+	
LH1G9				+		
LH2A5			+			
LH2A7			+			
LH2A9			+			
LH2A12			+			
LH2B4			+			
LH2B5			+			
LH2F8			+			
LH2F10		+				
LH2G5			+			
LH2G6			+			

LH2G10		+		
LH2H10		+		
LH2H12			+	
01F157				+
03F159				+
06C30			+	
08C32			+	+
08C128		+		
08D44				+
09C33			+	
09D45				+
09C129	+		+	
09H93			+	
10F70		+	+	
11B23			+	
12G180				+

Table S3. Antagonistic activity of 02C26, 09C129, LH1G9, and LG1D9 against CF-Ps.

	CP-Ps (AU strain No.)			
Env-P	*Achromobacter* spp.	*Burkholderia* spp.	*P. aeruginosa*	*Stenotrophomonas* spp.
02C26	33619	33602, 33589, 33586, 33568	18422, 27282	
09C129	33282, 33507	32976	12176, 15152, 17108, 18081, 27282, 30307	33211
LH1G9		33383, 33407, 33046, 33586, 33351, 33298	9276, 18422	
LG1D9		33383, 32976, 33589, 33586, 33602, 33046, 33298	9276, 8215, 12175, 12176, 15031, 15152, 15873, 17108, 17152, 17766, 18234, 20339, 30307	

CF-PAs shaded grey are MDR pathogens.

Table S4. Genome statistics of env-Ps 02C26, 09C129, LG1D9, and LH1G9.

	02C26		09C129		LG1D9		LH1G9	
	Number	% of total	Number	% of total	Number	% of total	Number	% of total
JGI Genome ID	2706794715	-	2703719185	-	2716884900	-	2740891816	-
Total bases	5404496	100	6897567	100	6289439	100	6548975	100
Coding bases	4875775	90.22	6108767	88.56	5595742	88.97	5821495	88.89
Total Genes	4959	100	6199	100	5707	-	6116	-
Protein coding genes	4811	97.02	6047	97.55	5562	97.46	5887	96.26
rRNA genes	148	2.98	152	2.45	19	0.33	19	0.31
tRNA genes	22	0.44	67	1.08	69	1.21	66	1.08
Protein coding genes:								
with function	3943	79.51	4981	80.35	4504	78.92	4853	79.35
without function	868	17.50	1066	17.20	1058	18.54	1034	16.91

with COGs	3637	73.34	4604	74.27	4129	72.35	4244	69.39
with Pfams	4212	84.94	5312	85.69	4812	84.32	5020	82.08
BGCs	3	2.06	13	4.52	7	3.22	7	2.73
Genes in BGCS	102	2.06	280	4.52	184	3.22	167	2.73

Table S5. Predicted ORFs for BGC 161819466 in strain 02C26.

ORF	JGI locus tag Ga0151585_	AA length	Predicted product	Best hit genome
1	111511	362	(R,R)-butanediol dehydrogenase / meso-butanediol dehydrogenase / diacetyl reductase	*Pseudomonas putida* GB-1
2	111512	182	adenine phosphoribosyltransferase	*Pseudomonas putida* KT2440
3	111513	244	transcriptional regulator, Crp/Fnr family	*Pseudomonas putida* KT2440
4	111514	460	coproporphyrinogen III oxidase, anaerobic	*Pseudomonas putida* S16
5	111515	227	hypothetical protein	*Pseudomonas putida* W619
6	111516	69	cytochrome oxidase maturation protein, cbb3-type	*Pseudomonas putida* W619
7	111517	820	Cu2+-exporting ATPase	*Pseudomonas entomophila* L48
8	111518	174	hypothetical protein	*Pseudomonas entomophila* L48
9	111519	471	cytochrome c oxidase accessory protein FixG	*Pseudomonas entomophila* L48

10	111520	326	cytochrome c oxidase cbb3-type subunit 3	*Pseudomonas entomophila* L48
11	111521	61	cytochrome c oxidase cbb3-type subunit 4	*Pseudomonas putida* W619
12	111522	202	cytochrome c oxidase cbb3-type subunit 2	*Pseudomonas putida* S16
13	111523	480	cytochrome c oxidase cbb3-type subunit 1	*Pseudomonas putida* GB-1
14	111524	313	cytochrome c oxidase cbb3-type subunit 3	*Pseudomonas putida* GB-1
15	111525	65	cytochrome c oxidase cbb3-type subunit 4	*Pseudomonas entomophila* L48
16	111526	202	cytochrome c oxidase cbb3-type subunit 2	*Pseudomonas putida* W619
17	111527	474	cytochrome c oxidase cbb3-type subunit 1	*Pseudomonas putida* W619
18	111528	230	hypothetical protein	
19	111529	94	hypothetical protein	*Pseudomonas entomophila* L48
20	111530	183	inhibitor of the KinA pathway to sporulation, predicted exonuclease	*Pseudomonas putida* KT2440

21	111531	275	amino acid ABC transporter substrate-binding protein, PAAT family	*Pseudomonas entomophila* L48
22	111532	176	RNA polymerase sigma-70 factor, ECF subfamily	*Pseudomonas entomophila* L48
23	111533	4317	non-ribosomal peptide synthase domain TIGR01720/amino acid adenylation domain-containing protein	*Pseudomonas entomophila* L48
24	111534	539	hypothetical protein	
25	111535	467	efflux transporter, outer membrane factor (OMF) lipoprotein, NodT family	*Pseudomonas entomophila* L48
26	111536	653	macrolide transport system ATP-binding/permease protein	*Pseudomonas putida* W619
27	111537	392	membrane fusion protein, macrolide-specific efflux system	*Pseudomonas putida* BIRD-1
28	111538	172	RNA polymerase sigma-70 factor, ECF subfamily	*Pseudomonas putida* W619
29	111539	159	response regulator receiver domain-containing protein	*Pseudomonas entomophila* L48
30	111540	444	arabinose efflux permease, MFS family	*Pseudomonas putida* KT2440

31	111541	554	electron-transferring-flavoprotein dehydrogenase	*Pseudomonas putida* KT2440
32	111542	249	electron transfer flavoprotein beta subunit	*Pseudomonas aeruginosa* C 1426
33	111543	309	electron transfer flavoprotein alpha subunit apoprotein	*Pseudomonas entomophila* L48
34	111544	273	amino acid ABC transporter substrate-binding protein, PAAT family	*Pseudomonas putida* F1
35	111545	118	hypothetical protein	*Pseudomonas putida* F1
36	111546	269	Outer membrane protein OmpA	*Pseudomonas entomophila* L48
37	111547	480	transcriptional regulator, GntR family	*Pseudomonas putida* KT2440
38	111548	164	hypothetical protein	
39	111549	84	hypothetical protein	
40	111550	202	START domain-containing protein	*Pseudomonas putida* F1
41	111551	429	citrate synthase	*Pseudomonas putida* F1

Table S6. Predicted ORFs for BGC 161819467 in strain 06C26.

ORF	JGI locus tag Ga0151585_	AA length	Predicted product	Best hit genome
1	111745	358	monosaccharide ABC transporter substrate-binding protein, CUT2 family	*Pseudomonas putida* GB-1
2	111746	37	hypothetical protein	
3	111747	72	hypothetical protein	*Pseudomonas putida* W619
4	111748	1235	methionine synthase (B12-dependent)	*Pseudomonas putida* F1
5	111749	769	fatty acid cis/trans isomerase	*Pseudomonas putida* W619
6	111750	344	peptidoglycan/LPS O-acetylase OafA/YrhL	*Pseudomonas entomophila* L48
7	111751	194	Fe/S biogenesis protein NfuA	*Pseudomonas putida* W619
8	111752	201	protein SCO1/2	*Pseudomonas putida* KT2440
9	111753	162	hypothetical protein	*Pseudomonas putida* KT2440
10	111754	75	hypothetical protein	

11	111755	277	transcriptional regulator, AraC family	*Pseudomonas putida* GB-1
12	111756	104	branched-chain amino acid transport protein	*Pseudomonas putida* GB-1
13	111757	233	4-azaleucine resistance probable transporter AzlC	*Pseudomonas putida* W619
14	111758	214	hypothetical protein	
15	111759	357	hypothetical protein	
16	111760	210	threonine/homoserine, lactone efflux protein	*Pseudomonas entomophila* L48
17	111761	267	hypothetical protein	*Pseudomonas putida* W619
18	111762	306	transcriptional regulator, LysR family	*Pseudomonas putida* S16
19	111763	786	DNA damage-inducible DNA polymerase II	*Pseudomonas putida* GB-1
20	111764	3083	non-ribosomal peptide synthase domain/amino acid adenylation domain-containing protein	*Pseudomonas fluorescens* SBW25
21	111765	352	Taurine dioxygenase, alpha-ketoglutarate-dependent	*Pseudomonas entomophila* L48
22	111766	1026	amino acid adenylation domain-containing protein	*Pseudomonas entomophila* L48

23	111767	3489	non-ribosomal peptide synthase domain TIGR01720/amino acid adenylation domain-containing protein	*Pseudomonas fluorescens* Pf0-1
24	111768	145	transposase, mutator family	*Pseudomonas brassicacearum brassicacearum* NFM421
25	111769	51	hypothetical protein	
26	111770	837	iron complex outer membrane receptor protein/outer-membrane receptor for ferric coprogen and ferric-rhodotorulic acid	*Achromobacter xylosoxidans* A8
27	111771	551	putative ATP-binding cassette transporter	*Pseudomonas putida* KT2440
28	111772	582	choline/carnitine/betaine transport	*Pseudomonas putida* W619
29	111773	338	protein N-acetyltransferase, RimJ/RimL family	*Pseudomonas putida* F1
30	111774	292	formylglycine-generating enzyme, required for sulfatase activity	*Pseudomonas brassicacearum brassicacearum* NFM421

31	111775	426	selenocysteine lyase/cysteine desulfurase	*Pseudomonas fluorescens* SBW25
32	111776	457	Zn-dependent dipeptidase	*Pseudomonas brassicacearum brassicacearum* NFM421
33	111777	187	hypothetical protein	*Pseudomonas putida* GB-1
34	111778	96	hypothetical protein	
35	111779	276	hypothetical protein	*Pseudomonas putida* S16
36	111780	239	hypothetical protein	*Pseudomonas entomophila* L48
37	111781	318	EamA domain-containing membrane protein RarD	*Pseudomonas entomophila* L48
38	111782	294	DNA-binding transcriptional regulator, LysR family	*Pseudomonas entomophila* L48
39	111783	389	diguanylate cyclase (GGDEF) domain-containing protein	*Pseudomonas fluorescens* Pf0-1
40	111784	469	hypothetical protein	
41	111785	301	protein phosphatase 2C	*Pseudomonas entomophila* L48

42	111786	796	outer-membrane receptor for ferric coprogen and ferric-rhodotorulic acid	*Pseudomonas protegens* Pf-5
43	111787	307	FecR family protein	*Pseudomonas protegens* Pf-5
44	111788	189	RNA polymerase sigma-70 factor, ECF subfamily	*Pseudomonas protegens* Pf-5
45	111789	173	RNA polymerase sigma-70 factor, ECF subfamily	*Pseudomonas putida* W619
46	111790	319	FecR family protein	*Pseudomonas putida* W619
47	111791	824	outer-membrane receptor for S5 and ferric-rhodotorulic acid	*Pseudomonas putida* W619
48	111792	48	hypothetical protein	
49	111793	402	predicted arabinose efflux permease, MFS family	*Pectobacterium carotovorum brasiliensis* PBR1692
50	111794	195	transcriptional regulator, TetR family	*Pectobacterium atrosepticum* SCRI1043
51	111795	327	hypothetical protein	*Pseudomonas protegens* Pf-5

52	111796	340	methionine synthase (B12-independent)	*Pseudomonas syringae pv. aesculi* NCPPB 3681
53	111797	224	Anti-sigma-K factor RskA	*Pseudomonas fluorescens* SBW25

Table S7. Predicted ORFs from BGC 161848994 in strain LH1G9.

ORF	JGI locus tag Ga0199208_	AA length	Predicted product	Best hit genome
1	112373	884	Type VI secretion system protein VasG	*Yersinia kristensenii* ATCC 33638
2	112374	163	Type VI secretion system secreted protein Hcp	*Providencia rustigianii* DSM 4541
3	112375	572	Outer membrane protein OmpA-like peptidoglycan-associated protein	*Pseudomonas putida* W619
4	112376	227	Type VI secretion system protein ImpK	*Pseudomonas putida* W619
5	112377	451	Type VI secretion system protein ImpJ	*Pseudomonas putida* W619
6	112378	512	Type VI secretion system protein ImpC	*Yersinia pseudotuberculosis* sv. O:1 IP32670
7	112379	167	Type VI secretion system protein ImpB	*Yersinia pestis* sv. Orientalis CO-92
8	112380	542	Hypothetical protein	*Pseudomonas fluorescens* SBW25
9	112381	454	Microsomal dipeptidase-like Zn-dependent dipeptidase	*Pseudomonas fluorescens* SBW25

10	112382	288	Formylglycine-generating enzyme required for sulfatase activity	*Pseudomonas fluorescens* SBW25
11	112383	275	Formyl transferase-like protein	*Pseudomonas fluorescens* Ps_77
12	112384	550	Putative ATP-binding cassette transporter	*Pseudomonas fluorescens* Ps_22
13	112385	810	Outer membrane receptor for ferric coprogen and ferric-rhodotorulic acid	*Pseudomonas fluorescens* Ps_77
14	112386	3495	Amino acid adenylation domain-containing protein	*Pseudomonas fluorescens* SBW25
15	112387	4677	Non-ribosomal peptide synthase protein (TIGR01720 family)/amino acid adenylation domain-containing protein	*Pseudomonas fluorescens* SBW25
16	112388	66	Hypothetical	-
17	112389	390	Predicted MFS family arabinose efflux permease	*Pseudomonas fluorescens* SBW25
18	112390	451	Putative MATE family efflux protein	*Pseudomonas fluorescens* Ps_22
19	112391	190	Hypothetical protein	*Pseudomonas fluorescens* Ps_40

20	112392	347	Amidase	*Pseudomonas fluorescens* SBW25
21	112393	115	Putative FmdB family regulatory protein	*Pseudomonas fluorescens* SBW25
22	112394	409	Formamidase	*Pseudomonas fluorescens* SBW25
23	112395	229	Amino acid/amide ABC transporter ATP-binding protein	*Pseudomonas brassicacearum brassicacearum* NFM421
24	112396	252	Urea transport system ATP-binding protein	*Pseudomonas brassicacearum brassicacearum* NFM421
25	112397	386	Amino acid/amide ABC transporter membrane protein	*Pseudomonas brassicacearum brassicacearum* NFM421
26	112398	305	Amino acid/amide ABC transporter membrane protein	*Pseudomonas brassicacearum brassicacearum* NFM421

27	112399	402	Amino acid/amide ABC transporter substrate-binding protein	*Pseudomonas brassicacearum brassicacearum* NFM421
28	112400	1125	Signal transduction histidine kinase	*Pseudomonas brassicacearum brassicacearum* NFM421
29	112401	232	LuxR family two component transcriptional regulator	*Pseudomonas fluorescens* SBW25
30	112402	370	Response regulator receiver modulated metal dependent phosphohydrolase	*Pseudomonas mendocina* NK-01
31	112403	953	PAS domain S-box-containing protein	*Azotobacter vinelandii* DJ, ATCC BAA-1303

Table S8. Predicted ORFs for BGC 161816930 in strain LE6C9.

ORF	JGI locus tag Ga0139558_	AA length	Predicted product	Best hit genome
1	115526	633	general secretion pathway protein D	*Pseudomonas aeruginosa* sv. O12 PA7
2	115527	494	type II secretion system protein E (GspE)	*Pseudomonas aeruginosa* sv. O12 PA7
3	115528	404	general secretion pathway protein F	*Pseudomonas aeruginosa* sv. O12 PA7
4	115529	144	general secretion pathway protein G	*Pseudomonas aeruginosa* sv. O12 PA7
5	115530	156	general secretion pathway protein H	*Pseudomonas aeruginosa* sv. O12 PA7
6	115531	124	general secretion pathway protein I	*Pseudomonas aeruginosa* sv. O12 PA7
7	115532	200	general secretion pathway protein J	*Pseudomonas aeruginosa* sv. O12 PA7
8	115533	290	general secretion pathway protein K	*Pseudomonas aeruginosa* sv. O12 PA7
9	115534	360	type II secretion system protein L (GspL)	*Pseudomonas aeruginosa* sv. O12 PA7
10	115535	156	general secretion pathway protein M	*Pseudomonas aeruginosa* sv. O12 PA7

11	115536	238	MOSC domain-containing protein YiiM	*Pseudomonas aeruginosa* sv. O12 PA7
12	115537	405	MFS transporter, FSR family, fosmidomycin resistance protein	*Pseudomonas protegens* Pf-5
13	115538	74	hypothetical protein	
14	115539	196	acyl homoserine lactone synthase	*Burkholderia graminis* C4D1M
15	115540	183	LuxR family transcriptional regulator/LuxR family transcriptional regulator, transcriptional activator of rhlAB and lasB	*Acinetobacter baumannii* ATCC 17978
16	115541	163	phenazine biosynthesis protein A/B	*Pseudomonas aeruginosa* sv. O12 PA7
17	115542	163	phenazine biosynthesis protein A/B	*Pseudomonas aeruginosa* PACS2
18	115543	400	3-deoxy-D-arabinoheptulosonate-7-phosphate synthase	*Pseudomonas aeruginosa* ATCC 33351
19	115544	207	isochorismate hydrolase	*Pseudomonas aeruginosa* PAP7
20	115545	637	phenazine biosynthesis protein phzE	*Pseudomonas aeruginosa* PAP7

21	115546	278	trans-2,3-dihydro-3-hydroxyanthranilate isomerase	*Pseudomonas aeruginosa* ATCC 33351
22	115547	222	pyridoxamine 5'-phosphate oxidase	*Pseudomonas aeruginosa* UCBPP-PA14
23	115548	610	asparagine synthase (glutamine-hydrolysing)	*Pseudomonas aeruginosa* DoWo1
24	115549	612	gamma-glutamyltransferase 1 threonine peptidase	*Pseudomonas protegens* Pf-5
25	115550	166	acetlytransferase (GNAT) domain-containing protein	*Pseudomonas protegens* Pf-5

Table S9. Predicted ORFs for BGC 161816936 in strain LE6C9.

ORF	JGI locus tag Ga0139558_	AA length	Predicted protein	Best hit genome
1	113519	329	peptide/nickel transport system ATP-binding protein	*Pseudomonas brassicacearum brassicacearum* NFM421
2	113520	323	peptide/nickel transport system ATP-binding protein	*Pseudomonas brassicacearum brassicacearum* NFM421
3	113521	240	NAD(P)-dependent dehydrogenase, short-chain alcohol dehydrogenase family	*Stigmatella aurantiaca* DW4/3-1
4	113522	89	hypothetical protein	
5	113523	320	regulator of nucleoside diphosphate kinase	*Pseudomonas protegens* Pf-5
6	113524	210	hypothetical protein	
7	113525	421	hypothetical protein	
8	113526	1490	RHS repeat-associated core domain-containing protein	*Burkholderia cepacia* 383
9	113527	123	REP element-mobilizing transposase RayT	*Rubrivivax benzoatilyticus* JA2

10	113528	189	DJ-1/PfpI family protein	*Chromobacterium violaceum* ATCC 12472
11	113529	375	condensation domain-containing protein	*Bacillus amyloliquefaciens* Campbell F, DSM 7
12	113530	333	surfactin synthase thioesterase subunit	*Bacillus amyloliquefaciens* Campbell F, DSM 7
13	113531	451	glycine hydroxymethyltransferase	*Bacillus amyloliquefaciens* Campbell F, DSM 7
14	113532	244	multimeric flavodoxin WrbA	*Streptomyces ghanaensis* ATCC 14672
15	113533	372	winged helix DNA-binding domain-containing protein	*Bacillus amyloliquefaciens* Campbell F, DSM 7
16	113534	1840	amino acid adenylation domain-containing protein	*Bacillus amyloliquefaciens* Campbell F, DSM 7
17	113535	441	hypothetical protein	

18	113536	57	hypothetical protein	*Bacillus amyloliquefaciens* Campbell F, DSM 7
19	113537	474	arabinose efflux permease, MFS family	*Bacillus amyloliquefaciens* Campbell F, DSM 7
20	113538	442	condensation domain-containing protein	*Pseudomonas protegens* Pf-5
21	113539	456	hypotheical membrane protein	*Pseudomonas protegens* Pf-5
22	113540	510	glycosyl transferases group 1	
23	113541	329	Hypothetical protein	*Pseudomonas protegens* Pf-5
24	113542	451	PelD GGDEF domain-containing protein	
25	113543	173	hypothetical protein	*Pseudomonas protegens* Pf-5
26	113544	1196	tetratricopeptide repeat-containing protein	*Pseudomonas protegens* Pf-5
27	113545	936	hypothetical protein	*Pseudomonas protegens* Pf-5
28	113546	310	UDP-glucose 4-epimerase	*Pseudomonas protegens* Pf-5

29	113547	279	5'-nucleotidase, lipoprotein e(P4) family	
30	113548	545	hypothetical protein	
31	113549	66	hypothetical protein	Methanosarcina mazei Go1
32	113550	449	von Willebrand factor type A domain-containing protein	*Pseudomonas fluorescens* WH6
33	113551	525	alkaline phosphatase D	

Table S10. Predicted ORFs in strain LG1D9.

ORF	JGI locus tag Ga0172616_	AA length	Predicted product	Best hit genome
1	112982	573	ATP-binding cassette, subfamily B	*Pseudomonas fluorescens* ATCC 17400
2	112983	581	ATP-binding cassette, subfamily B	*Pseudomonas fluorescens* ATCC 17400
3	112984	204	DNA-binding transcriptional regulator, MarR family	*Pseudomonas fluorescens* ATCC 17400
4	112985	309	Acetyltransferase (GNAT) family protein	*Pseudomonas* sp. LE6C6
5	112986	288	Methyltransferase domain-containing protein	*Pseudomonas* sp. LE6C6
6	112987	545	FAD binding domain-containing protein	*Pseudomonas fluorescens* ATCC 17400
7	112988	254	3-methyladenine DNA glycosylase AlkC	*Pseudomonas fluorescens* ATCC 17400
8	112989	213	Peroxiredoxin	*Pseudomonas* sp. LE6C6
9	112990	556	Dehydrogenase (flavoprotein)	*Pseudomonas* sp. LE6C6

10	112991	493	Fatty acid amide hydrolase 2	*Pseudomonas fluorescens* ATCC 17400
11	112992	445	Transporter, CPA2 family	*Pseudomonas* sp. LE6C6
12	112993	208	Nicotinamidase-related amidase	*Pseudomonas* sp. LE6C6
13	112994	229	Nicotinamidase-related amidase	*Pseudomonas* sp. LE6C6

Table S11. Top five BGC hits per strain from NCBI BLAST analysis.

Strain / JGI BGC ID#	NCBI hit	Query coverage	E-value	Identity	Accession
02C26 / 161819466	*Pseudomonas putida* KT2440	95%	0	82%	NC_002947.4
	Pseudomonas monteilii strain USDA-ARS-USMARC-56711	59%	0	80%	NZ_CP013997.1
	Pseudomonas putida strain PA14H7	84%	0	80%	NZ_KN639176.1
	Pseudomonas citronellolis strain SJTE-3	68%	0	77%	NZ_CP015878.1
	Pseudomonas aeruginosa PAO1	63%	0	77%	NC_002516.2
02C26 / 161819467	*Pseudomonas plecoglossicida* strain XSDHY-P	28%	0	85%	NZ_CP031146.1
	Pseudomonas monteilii strain FDAARGOS_171	31%	0	85%	NZ_CP014062.1
	Pseudomonas monteilii strain B5	29%	0	85%	NZ_CP022562.1

	Pseudomonas sp. LTGT-11-2Z	29%	0	85%	NZ_CP033104.1
	Pseudomonas sp. Leaf58	35%	0	84%	NZ_CP032677.1
09C129 / 161816930	*Pseudomonas chlororaphis* subsp. chlororaphis strain DSM 50083	100%	0	98%	NZ_CP027712.1
	Pseudomonas chlororaphis strain ATCC 17415	91%	0	97%	NZ_CP027714.1
	Pseudomonas chlororaphis strain TAMOak81	99%	0	96%	NZ_CP027713.1
	Pseudomonas chlororaphis subsp. piscium strain ToZa7	99%	0	95%	NZ_CP027739.1
	Pseudomonas chlororaphis subsp. piscium strain ChPhzTR44	99%	0	95%	NZ_CP027711.1
09C129 / 161816936	*Pseudomonas chlororaphis* strain TAMOak81	78%	0	97%	NZ_CP027713.1

	Pseudomonas chlororaphis subsp. chlororaphis strain DSM 50083	69%	0	99%	NZ_CP027712.1
	Pseudomonas chlororaphis subsp. piscium strain ZJU60	40%	0	94%	NZ_CP027656.1
	Pseudomonas chlororaphis subsp. piscium strain DSM 21509	43%	0	94%	NZ_CP027707.1
	Pseudomonas chlororaphis subsp. piscium strain PCL1391	57%	0	94%	NZ_CP027736.1
LG1D9	*Pseudomonas fluorescens* strain PF08	99%	0	95%	NZ_CP032618.1
	Pseudomonas fluorescens strain UK4	100%	0	91%	NZ_CP008896.1
	Pseudomonas sp. St29	87%	0	77%	NZ_AP014628.1
	Pseudomonas sp. Os17	85%	0	77%	NZ_AP014627.1
	Pseudomonas monteilii strain B5	73%	0	74%	NZ_CP022562.1

LH1G9 / 161848994	*Pseudomonas veronii* 1YdBTEX2	99%	0	99%	LT599583.1
	Pseudomonas veronii strain R02	99%	0	99%	NZ_CP018420.1
	Pseudomonas sp. R5-89-07	57%	0	84%	NZ_CP027727.1
	Pseudomonas sp. R1-43-08	56%	0	84%	NZ_CP027734.1
	Pseudomonas sp. R4-39-08	56%	0	84%	NZ_CP027733.1

Strain	pfam03781	pfam00664	pfam01244	pfam00975	pfam07715	pfam00593	pfam07690	pfam00440	pfam13561	pfam01370	pfam01965	pfam06224	pfam00464	pfam13434	pfam02668	pfam00266	pfam12833	pfam07660
	Sulfatase-modifying enzyme	ABC transporter transmembrane region	Membrane dipeptidase (Peptidase family M19)	Thioesterase domain	TonB-dependent Receptor Plug Domain	TonB dependent receptor	Major Facilitator Superfamily	Bacterial regulatory proteins- tetR family	Enoyl-(Acyl carrier protein) reductase	NAD dependent epimerase/dehydratase family	DJ-1/PfpI family	Winged helix DNA-binding domain	Serine hydroxymethyltransferase	L-lysine 6-monooxygenase (NADPH-requiring)	Taurine catabolism dioxygenase TauD- TfdA family	Aminotransferase class-V	Helix-turn-helix domain	Secretin and TonB N terminus short domain
Pseudomonas sp. LH1G9	1	1	1	1	1	1	1	0	0	0	0	0	0	0	0	0	0	0
Pseudomonas veronii 1YdBTEX2	1	1	1	1	1	1	1	0	0	0	0	0	0	0	0	0	0	0
Pseudomonas veronii strain R4 Genome sequencing	1	1	1	1	1	1	1	0	0	0	0	0	0	0	0	0	0	0
Pseudomonas panacis BS2778	1	1	1	1	1	1	1	0	0	0	0	0	0	0	0	0	0	0
Pseudomonas veronii DSM 11331	1	1	1	1	1	1	1	0	0	0	0	0	0	0	0	0	0	0
Pseudomonas sp. GV062	1	1	1	1	1	1	1	0	0	0	0	0	0	0	0	0	0	0
Pseudomonas sp. GV058	1	1	1	1	1	1	1	0	0	0	0	0	0	0	0	0	0	0
Pseudomonas sp. GV101	1	1	1	1	1	1	1	0	0	0	0	0	0	0	0	0	0	0
Pseudomonas sp. GV082	1	1	1	1	1	1	1	0	0	0	0	0	0	0	0	0	0	0
Pseudomonas sp. GV077	1	1	1	1	1	1	1	0	0	0	0	0	0	0	0	0	0	0
Pseudomonas sp. GV086	1	1	1	1	1	1	1	0	0	0	0	0	0	0	0	0	0	0
Pseudomonas fluorescens Ps006	1	1	1	1	1	1	1	0	0	0	1	0	0	0	0	1	2	1
Pseudomonas fluorescens H21	1	1	1	1	1	1	1	1	0	0	0	0	0	0	0	1	0	0
Pseudomonas antarctica PAMC 27494	1	1	1	1	1	1	1	1	0	0	0	0	0	0	0	1	0	0
Pseudomonas fluorescens DSM 50090	1	1	1	1	1	1	1	1	0	0	0	0	0	0	0	1	0	0
Pseudomonas fluorescens ATCC 13525	1	1	1	1	1	1	1	1	0	0	0	0	0	0	0	1	0	0
Pseudomonas extremaustralis DSM 17835	1	1	1	1	2	2	1	0	0	0	0	0	0	0	0	0	1	0
Pseudomonas fluorescens GcM5-1A	1	1	1	1	3	1	2	1	1	0	2	0	0	1	1	1	2	2
Pseudomonas fragi P121	0	1	0	2	2	2	3	0	0	0	0	0	0	1	2	0	0	1
Pseudomonas alcaligenes MRY13-0052	1	1	1	2	1	1	1	0	0	0	0	0	0	1	1	1	0	1
Pseudomonas sp. StFLB209	1	1	1	1	2	2	0	0	0	1	0	0	0	1	1	0	0	1
Pseudomonas moraviensis Devor	1	1	1	1	1	1	2	0	1	0	2	0	0	1	1	1	1	1
Pseudomonas fluorescens AU20219	1	1	1	1	1	1	2	1	1	1	2	0	0	1	1	1	1	1
Pseudomonas sp. 311C	1	1	1	1	1	1	2	1	1	1	2	0	0	1	1	1	1	1
919A	1	1	1	1	1	1	2	1	1	1	2	0	0	1	1	1	1	1
Pseudomonas fluorescens AU13852	1	1	1	1	1	1	2	1	1	1	2	0	0	1	1	1	1	1
Pseudomonas sp. Os17	1	1	1	1	1	1	2	1	0	1	2	0	0	1	1	1	1	1
Pseudomonas saponiphila DSM 9751	1	1	1	1	1	1	2	1	0	1	2	0	0	1	1	1	1	1

[illegible]

Figure S1. Heat map of Pfams that show similarity to BGCs in strains 02C26, LH1G9, 09C129, and LG1D9. The 95 most similar BGCs were identified in n JGI ABC and used to show similarities and differences within each cluster. The JGI ABC Pfam number and predicted products are listed at the top X-axis. The heat map represents the number of Pfams in a BGC and range from 0-5. The numbers correspond to colors in Figure 5: yellow, 0 Pfams; lightest green, one Pfam to darkest green, 5 Pfams. The left Y-axis consists of strains that represent the phylogeny in Figure 5.
